# Biomechanical consequences of the intervertebral disc centre of rotation kinematics during lateral bending and axial rotation

**DOI:** 10.1038/s41598-023-29551-7

**Published:** 2023-02-23

**Authors:** Roman Allais, Antoine Capart, Anabela Da Silva, Olivier Boiron

**Affiliations:** 1 CNRS, Centrale Marseille, IRPHE, Aix Marseille Univ, 13013 Marseille, France; 2 Aix Marseille Univ, CNRS, Centrale Marseille, Institut Fresnel, 13013 Marseille, France

**Keywords:** Cartilage, Mechanical engineering, Biomedical engineering

## Abstract

The location of the instantaneous centre of rotation (ICR) of a lumbar unit has a considerable clinical importance as a spinal health estimator. Consequently, many studies have been conducted to measure or estimate the ICR during rotations in the three anatomical planes; however the results reported are widely scattered. Even if some inter-subjects variability is to be expected, such inconsistencies are likely explained by the differences in methods and experiments. Therefore, in this paper we seek to model three behaviours of the ICR during lateral bending and axial rotation based on results published in the literature. In order to assess the metabolic and mechanical sensibility to the assumption made on the ICR kinematics, we used a previously validated three dimensional non-linear poroelastic model of a porcine intervertebral disc to simulate physiological lateral and axial rotations. The impact of the geometry was also briefly investigated by considering a 11$^\circ$ wedge angle. From our simulations, it appears that the hypothesis made on the ICR location does not significantly affect the critical nutrients concentrations but gives disparate predictions of the intradiscal pressure at the centre of the disc (variation up to 0.7 MPa) and of the displacement fields (variation up to 0.4 mm). On the contrary, the wedge angle does not influence the estimated intradiscal pressure but leads to minimal oxygen concentration decreased up to 33% and increased maximal lactate concentration up to 13%. While we can not settle on which definition of the ICR is more accurate, this work suggests that patient-specific modeling of the ICR is required and brings new insights that can be useful for the development of new tools or the design of surgical material such as total lumbar disc prostheses.

## Introduction

The position of the instantaneous centre of rotation (ICR) of a functional lumbar unit is a known metric of the spinal health and stability^1,2^. In the past decades, extensive research has been conducted on the estimation of the ICR location during full range physiological motions but the results of these studies are surprisingly scattered. Ogston and colleagues, in their preliminary study^3^, described how to measure the centrode length (i.e. ICR trajectory) during flexion-extension in a clinical setting using radiographs. Meanwhile, Gertzbein et al. reported that intervertebral disc (IVD) degeneration at the L4/L5 level significantly impacts the length and pattern of the centrode during the same motion^2^. More specifically, their results suggest that early stages of degeneration seem to increase the centrode length while higher stages decrease it. However, ICR location estimation through radiographs appears to be subject to considerable errors for rotation less than 5$^\circ$ in the sagittal plane, which raises the question of using an average centre of rotation instead^4^.

**Table 1:** Summary of studies on the ICR location during lateral bending and axial rotation.

Reference	Motion	Study type	Population	ICR location
Dimnet et al.^5^	LB	In vivo	20 male subjects, 20–40 years old, levels T11–L5	IVD centre
Rolander^6^	LB	Ex vivo	38 cadavers (13 F/25 M), 4–76 years old, levels L1–L5	Superior, opposite side of the rotation
White and Panjabi^7^	LB	N/A	N/A	Opposite side of the rotation
Rousseau et al.^8^	LB	Ex vivo	12 cadavers (4 F/8 M), 50–64 years old, level L5/S1	Moving towards the side of the bending
Schmidt et al.^9^	LB	In silico	Finite-elements model of a L4/L5 level	IVD centre and moving towards the side of the bending
Cossette et al.^10^	AR	Ex vivo	12 cadavers, level L3/L4	Posterior side, moving towards the side of the rotation
White and Panjabi^7^	AR	N/A	N/A	IVD centre
Schmidt et al.^9^	AR	In silico	Finite-elements model of a L4/L5 level	IVD centre and moving posteriorly, towards the right side for an axial rotation to the left

Regarding lateral bending (LB) of the lumbar spine, the average centre of rotation for right to left lateral bending appears to be located near the centre of the discs^5^. On the other hand, when studying right and left lateral bending separately two average centres of rotation are described in the study of Rolander^6^. Although the results are scattered, Rolander measured the average centre of rotation in the superior contralateral part of the disc (e.g. right side of the disc for left side bending), which was later corroborated by other researchers^7^. Nevertheless those studies only estimated an average centre of rotation. More recent works gave some insights on the motion of the ICR during lateral bending: for example, an upward shift towards the side of the bending for the L5/S1 joint was reported^8^. Finally, the numerical study of Schmidt and colleagues supported to some extent these findings as their finite elements model predicted the same trend for a L4/L5 joint^9^.

As for twisting (sometimes referred to as axial rotation (AR)), although not providing with numerical data, Cossette et al. measured the ICR in the posterior part of the disc with a trend of moving towards the side of the rotation with increasing rotation^10^. Quantitative results that refined the findings of Cossette and colleagues can be found in numerical studies: using a finite element model, a team of researchers reported the ICR trajectory as a function of increasing torque^9^. However, these results do not match the ones illustrated elsewhere where the ICR is displayed at the centre of the disc^7^.

While some of the variance in the ICR estimations found in the literature can be explained by inter-subjects variability, it should be borne in mind that the apparatus and experimental conditions used in the cited literature are diverse. One can indeed argue that results from in vivo, ex vivo and in silico studies can only be compared with care. The methods and key findings from the cited literature concerning the ICR locations during lateral bending and axial rotation are summed up in Table 1.

The estimation of the ICR of lumbar spine units recently received a renewed interest with the development of musculoskeletal models^11,12^. Musculoskeletal modeling can be used to help planning the surgery which will yield the best biomechanical outcomes; yet, the inverse dynamics resolution used in such models depends on the validity and modeling of the prescribed kinematics. A sensitivity study of the outcomes of a L4/L5 total disc replacement pointed out that a 5mm sagittal shift of the centre of rotation considerably impacts the predicted joint resultant, ligament and muscle forces during flexion-extension simulations^11^. Senteler et al. refined the analysis with their parametric approach on the whole lumbar spine: they predicted that at 30$^\circ$ flexion the anterior shift of the ICR decreases the compression forces at a rate of 2.5%/mm^12^. A proper understanding and an adequate estimation of the ICR location is therefore of prime interest for both clinical and modeling applications.

To get a right estimation of the ICR, the kinematics should be as close as possible to the physiological reality. There are two ways of modeling a rotation: through an applied moment^9,13,14^ or a prescribed displacement^15^. We deem the latter to better apprehend the physiology as it enforces the coupled rotations reported in the in vivo studies^16–18^ and it allows to specify the range of motion. On the other hand, with a moment applied, even though it would show the true kinematics of our disc, the range of motion can not be known beforehand and the ICR kinematics can not easily be constrained. As the goal of the study is to compare ICR behaviours reported in the literature, the kinematics of this specific disc are not of particular relevance.

Faced with the inconsistencies in the ICR locations and kinematics modeling, the aim of this paper is to provide with quantitative results on the ICR behaviour during lateral bending and axial rotation. Previous work has been published on the sensitivity of some biomechanical parameters to the position of an average centre of rotation during flexion/extension^11,12^. However, they made the convenient but non-physiological assumption of a fixed centre of rotation for their simulations. Others presented finite elements models, validated for LB and AR, but without considering their sensitivity to the ICR location^19,20^. Finally, drawing meaningful comparisons between different numerical studies can be delicate as the anatomic completeness among the models varies. Also, we tried to tackle this issue by comparing the outputs of our previously validated porcine IVD model to its modified version which mimics the geometry of a L4/L5 human disc.

Hence, we seek here to assess the variation on both the mechanical and metabolic features of the IVD caused by the hypothesis made on the ICR kinematics. We considered 3 different locations of centre of rotation during lateral bending and axial rotation reported in the previous literature and we defined them in our finite element model of an IVD. For the sake of brevity, since less studies seem to have been conducted on the ICR kinematics during lateral bending and axial rotation, we chose to focus on those motions while flexion and extension could be the topic of a future study.

**Figure 1:**
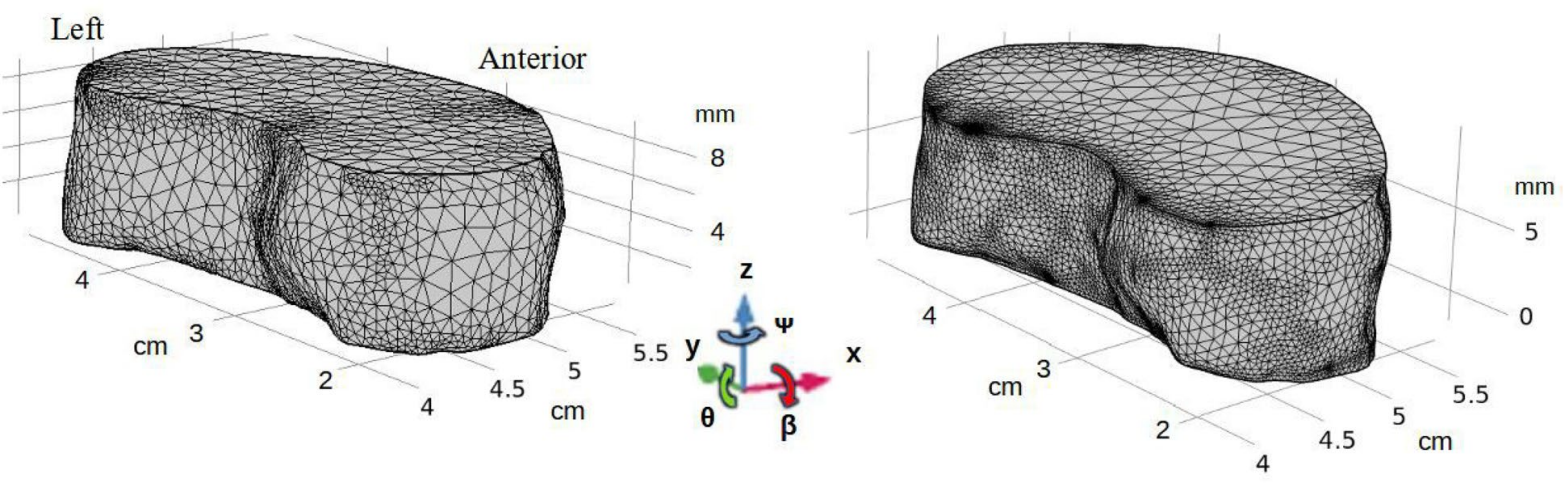
Two models of the intervertebral disc with their final mesh: initial geometry (left) and modified geometry (right). *x* axis points anteriorly, *y* points left and *z* points superiorly.

## Methods

### Finite elements model

We used a previously published multiphysics three-dimensional nonlinear poroelastic model of a porcine lumbar intervertebral disc^21,22^. Succinctly, the geometry and porosity field in an unloaded state of the 4-month-old porcine disc were obtained using quantitative MRI using a 4.7-T horizontal superconducting magnet of a 47/30 Biospec Avance system (Bruker, Germany), equipped with a Bruker 120-mm BGA12SL (200 mT/m) gradient insert, using a proton RF coil (RF RES 200 1H Model 1PT5346; Bruker BioSpin MRI GmbH, Germany). The slice thickness was 0.75 mm in the transverse plane and 1 mm in the sagittal plane^21^ and we used multi-slices multi-echos sequences^23^. The main interest of this approach is to quantitatively and accurately retrieve the main internal biomechanical variables of the disc (i.e. the porosity field and the geometry). Then, the IVD was modeled by a biphasic fully saturated porous medium^24^ with a swelling pressure^25^ and a fiber-reinforced extracellular matrix to account for the anisotropy of the collagen network in the annulus fibrosus (AF)^26^. The nucleus pulposus (NP), annulus fibrosus and cartilaginous endplates (CEP) were segmented using the porosity field as a proxy to identify the different anatomical compartments using the software Mimics 14.1 (Materialise NV, Leuven, Belgium). All the segmentations were performed by one operator to minimise the reconstruction errors (estimated to less than 3.5% on the disc volume). Each of these compartments had their own hyperelastic constitutive law that were fit to the measurements of relaxation tests made on the actual disc using an optimisation scheme and a transition area where all the physical parameters are smoothed^22^. This mechanical model is then coupled with a metabolic model^27^ to predict the concentration fields of oxygen and lactate in response to a deformation; the glucose concentration can be derived from the energy metabolism equation assuming a fully glycolytic pathway. Pictures of the experimental study can be found in a previous publication^23^ and the complete derivation of the model as well as the couplings between the metabolism and the deformation were already published^22,28^ but a summary of the main mechanical properties are recalled in Supplementary Table 1. However, human intervertebral discs display a wedge angle^29,30^, whereas our initial geometry does not. Therefore, we also performed simulations on a modified version of the original model to be closer to a human disc and to examine the sensitivity to the geometry. Using a CAD software, we modified the cartilaginous endplates to create a 11$^\circ$ wedge angle to mimic the geometry of a L4/L5 human disc^30^. Removing materials would have further stray away our model from a human disc, that is why we chose to add materials instead. As a result, the posterior height was kept the same resulting in a 2.6mm increase in the anterior height and in a 7% increase of the disc volume. All the added volume was given the same properties as the original cartilaginous endplates; it should be noted that this assumption could lead to an oversized height of the endplate on the most anterior side but we do not think it would significantly alter our results as the cartilaginous endplates are considerably stiffer than the nucleus and annulus and are therefore less prone to deformation. Both the original and modified geometry can be seen on Fig. 1 and more details on the meshes will be provided at the end of the section.

### Validation

The validation of our initial model against experimental uniaxial relaxation tests was described in previous studies^21,22^. The validation is extended to lateral bending and axial rotation through a methodical comparison with previous models. Our results on the intradiscal pressure, displacement and concentration fields stay in the range of the values we observed during our experimental validation and were additionally compared with results from in vivo, in vitro and in silico studies.

### Initial state

The imaging approach allows to recover the geometry and porosity field of the swollen disc at the physiological equilibrium. In order to retrieve the osmotic pressure and concentration fields corresponding to the imaged unloaded disc at this equilibrium, we ran a stationary boundary values problem. For the mechanical problem, no displacement is applied on the IVD and its lateral face is free to swell as a consequence of the osmotic pressure. For the nutrients diffusion problem, Dirichlet conditions are set on the three boundaries using the concentration values found in the plasma surrounding a human disc, as usually used in IVD finite-element models^31–33^. The output of this simulation is then used as initial values for our dynamic simulations. All the simulations were performed using *COMSOL Multiphysics 5.5* on a workstation with a Intel(R) Xeon(R) W-11955M CPU and 64GB of RAM.

### Motion description

#### Range of motion and displacement

For both LB and AR, we chose to model the rotations by a prescribed displacement of the superior endplate while the inferior remains rigidly fixed. Thus, it makes possible to impose the coupled rotations and models a more physiological displacement. The displacement **u** underwent by the superior endplate is defined by Eq. (1). As for the amplitudes of rotations, we used the ranges of motion measured by Pearcy and Tibrewal^16^. Briefly, they asked their subjects to perform full-range lateral and axial bending while they were taking biplanar radiographs to notably measure the three dimensional rotation between each lumbar level. The specific results for a L4/L5 disc we retained are reported in Table 2. Finally, in the vein of mimicking as close as possible a real motion, we smoothed the displacement over 4 s. At $t=0$ s, the disc is in an unloaded state, at $t=2$ s the maximal values presented in Table 2 are reached and at $t=4$ s the disc is back in its initial state.

**Table 2:** Prescribed rotations in the anatomical planes for LB and AR simulations.

Motion	Frontal ($\beta$)	Transverse ($\Psi$)	Sagittal ($\Theta$)
Lateral bending	2.5$^\circ$	1$^\circ$	$-0.5^\circ$
Axial rotation	1.5$^\circ$	1.5$^\circ$	0

Source: Pearcy and Tibrewal^16^.

1 $$\begin{aligned} \begin{pmatrix} u_{x} \\ u_{y} \\ u_{z} \\ \end{pmatrix} = \begin{pmatrix} cos\theta cos\Psi \, &{} sin\beta sin\theta cos\Psi -cos\beta sin\Psi \, &{} sin\beta sin\Psi + cos\beta sin\theta cos\Psi \\ cos\theta sin\Psi \, &{} cos\beta cos\Psi +sin\beta sin\theta sin\Psi \, &{} cos\beta sin\theta sin\Psi -sin\beta cos\Psi \\ -sin\theta \, &{} sin\beta cos\theta \, &{} cos\beta cos\theta \\ \end{pmatrix} \begin{pmatrix} x - c_{x}\\ y - c_{y} \\ z - c_{z} \\ \end{pmatrix} - \begin{pmatrix} x - c_{x}\\ y - c_{y} \\ z - c_{z} \\ \end{pmatrix} \end{aligned}$$

With $\beta$, $\theta$ and $\Psi$ as defined on Fig. 1.

#### Lateral bending

**Figure 2:**
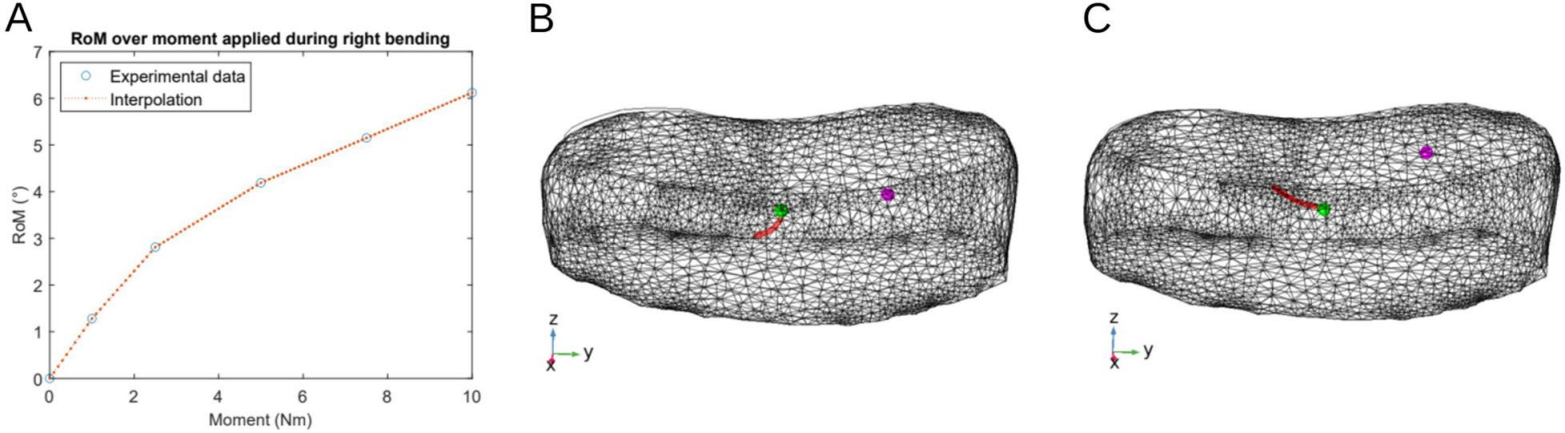
(**A**) Range of motion (RoM) over moment applied in the frontal plane for a L4/L5 human disc during lateral bending. Experimental values taken from Heuer et al.^13^. (**B**) Positions and trajectories of the 3 ICR definitions during LB. Green: hypothesis LB-1. Magenta: hypothesis LB-2. Red: hypothesis LB-3. (**C**) Positions and trajectories of the 3 ICR definitions during AR. Green: hypothesis AR-1. Magenta: hypothesis AR-2. Red: hypothesis LB-3.

From there on, we define new variables: $bar_{x,y,z}$ and $c_{x,y,z}$ respectively stand for the spatial coordinates of the IVD barycentre and of the centre of rotation considered; $D_{sag}$, $D_{coro}$ and *H* are the lengths of the IVD in respectively the antero-posterior, lateral and axial direction, passing through its barycentre. From the literature review displayed in Table 1, we kept three different definitions of the L4/L5 centre of rotation to implement them in our model:

1. A centre of rotation set at the centre of the disc^5^:
  2 $$\begin{aligned} \left\{ \begin{array}{lll} c_{x} = bar_{x} \\ c_{y} = bar_{y} \\ c_{z} = bar_{z} \\ \end{array} \right. \end{aligned}$$
2. A centre of rotation fixed in the posterior, left side of the IVD for a right side bending^6,15^:
  3 $$\begin{aligned} \left\{ \begin{array}{lll} c_{x} &{}= bar_{x} - \frac{D_{sag}}{4} \\ c_{y} &{}= bar_{y} + \frac{D_{coro}}{5} \\ c_{z} &{}= bar_{z} \\ \end{array} \right. \end{aligned}$$
3. A moving ICR, starting from the centre of the disc and moving towards the side of the bending^9^. However, Schmidt and colleagues reported the trajectory as a function of the increasing moment applied on the upper endplate of the L4 vertebra.
  We therefore referred to the experimental data they used to calibrate their model^13^. These data, which are reported in Fig. 2A, give the relationship between the moment applied on the upper endplate of the L4 vertebra and the range of motion (RoM) of the L4/L5 segment. Finally, using this approach, we could model the trajectory of the ICR as a function of the angle in the frontal plane. These three hypotheses are illustrated on Fig. 2B.

#### Axial rotation

For the axial rotation, we had the same approach as for LB: we kept three different definitions of the L4/L5 centre of rotation reported in previous works:

1. A centre of rotation set at the disc barycentre^7^:
  4 $$\begin{aligned} \left\{ \begin{array}{lll} c_{x} = bar_{x} \\ c_{y} = bar_{y} \\ c_{z} = bar_{z} \\ \end{array} \right. \end{aligned}$$
2. A centre of rotation in the superior ipsilateral side of the rotation^15^:
  5 $$\begin{aligned} \left\{ \begin{array}{lll} c_{x} &{}= bar_{x} - \frac{D_{sag}}{4} \\ c_{y} &{}= bar_{y} + \frac{D_{coro}}{5} \\ c_{z} &{}= H - h_{CEP} \\ \end{array} \right. \end{aligned}$$
  With $h_{CEP}$ the height of the superior cartilaginous endplate, which is 1.5 mm in our model.
3. A moving ICR, starting from the centre of the disc and moving posteriorly in the right side of the disc^9^. Just as for LB, we used the calibration data^13^ to match the RoM to an equivalent moment in order to compute the trajectory in the transverse plane described by Schmidt and coworkers^9^. These three hypotheses are illustrated on Fig. 2C.

### Mesh convergence

We performed a mesh convergence analysis to ensure mesh independence. By their off-centre locations, the centre of rotation kinematics as defined by hypotheses LB-2 and AR-2 are more likely to yield higher deformations and perturbations of both the mechanical and metabolic variables. We will therefore perform our mesh analysis on these specific kinematics for the initial and modified geometries.

Such a validation had already been done for our model for an uniaxial compression of the superior endplate^21,28^. For the two geometries we built three meshes of tetrahedral elements whose resolutions are detailed in Table 3; Lagrange quadratic interpolation elements were used for the displacement field and linear interpolation elements were used for the pressure and concentrations. The choice of the optimal mesh for each geometry was based on a trade-off between computation time and relative errors on the displacement, porosity, concentrations and total pressure fields, the latter being defined as the sum of the osmotic and interstitial pressure fields.

**Table 3:** Resolution of the considered meshes.

Initial geometry				Modified geometry			
Mesh	Elements	D.o.F.	Min. element size	Mesh	Elements	D.o.F.	Min. element size
Mesh 1	55,486	300,737	0.05 mm	Mesh 4	83,808	492,398	0.25 mm
Mesh 2	20,197	133,077	0.10 mm	Mesh 5	56,568	342,699	0.33 mm
Mesh 3	12,678	85,808	0.33 mm	Mesh 6	28,877	183,138	0.59 mm

*D.o.F*.: degrees of freedom.

### Anisotropic strain energy

The consequences on the strain energy were also investigated. More specifically, we looked at the temporal variation of the anisotropic strain energy normalised by the disc volume and its proportion with respect to the total energy. Briefly, we define in the annulus two fibre families according to their mean orientation with respect to the transverse plane in order to model the anisotropy of the collagen fibres network. Then, two anisotropic strain energy densities, namely $W_{anis1}$ and $W_{anis2}$, are associated with each family and their sum represents the total anisotropic strain energy density; the details of the derivation was previously described^28^ and the derivation is briefly recalled in Supplementary information 1. Finally, we computed the second Piola-Kirchhoff (PK) tensors associated with the anisotropic strain energy density ($PK_{aniso}$) and the one associated with the isotropic strain energy density ($PK_{iso}$). To investigate spatial variations as well, we looked at the ratio of the first eigenvalue of $PK_{aniso}$ over the first eigenvalue of $PK_{iso}$ at $t=2 \ s$.

### Peclet number

To draw a quantitative comparison between advection and diffusion, we computed the Peclet number associated with the oxygen and lactate. Half the posterior height of the disc was chosen as the characteristic diffusion length and the fluid velocity was derived using Darcy’s law for a porous medium. Consequently, a global Peclet number was computed at each timestep using the spatial average of the fluid velocity.

## Results

### Mesh convergence

**Figure 3:**
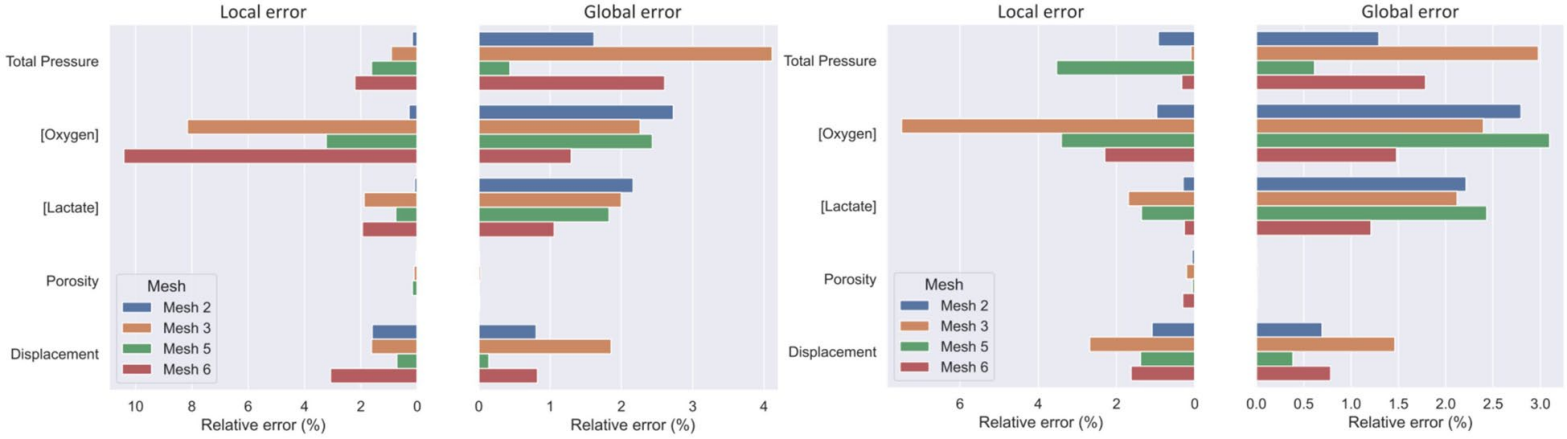
Mesh analysis for LB (left) and AR (right) at the full range of motion. [.] is used to express the concentration.

To assess the mesh convergence, we took Mesh 1 and Mesh 4 as references and used two metrics to assess the numerical accuracy of the four other meshes. The relative error on the average value over the whole disc was used as a global metric and the relative error on the value at the centre of the disc as a local metric. The results are displayed on Fig. 3. The total pressure field is the variable which yields the highest average relative error, up to 4% computed when using Mesh 3. On the other hand, when looking at the local metric, the nutrients concentration shows the highest relative error, up to 10% for the oxygen on Mesh 6. Because of these large errors on local values, meshes 3 and 6 were considered not fine enough and discarded. Finally, considering their overall acceptable relative errors ($<4\%$) and computation time, Mesh 2 and Mesh 5 were kept.

### Initial state

This conditioning step gives an average total pressure over the entire disc of 0.102 MPa with an increasing gradient as we move towards the centre of IVD. This is due to the higher concentration of fixed charges which consequently results in a higher osmotic pressure in the nucleus pulposus where the pressure reaches 0.203 MPa. The average oxygen concentration is $0.034 \ \textrm{mol} \, \textrm{m}^{-3}$ with a maximal value of $0.060 \ \mathrm{mol \, m}^{-3}$ found at the boundaries of the AF and a minimal value of $0.014 \ \mathrm{mol \, m}^{-3}$ inside the NP. The lactate concentration shows the opposite trend: values range from $0.9 \ \mathrm{mol \, m}^{-3}$ at the external borders of the AF to $4.12 \ \mathrm{mol \, m}^{-3}$ at the centre of the disc with an average of $2.566 \ \mathrm{mol \, m}^{-3}$.

**Figure 4:**
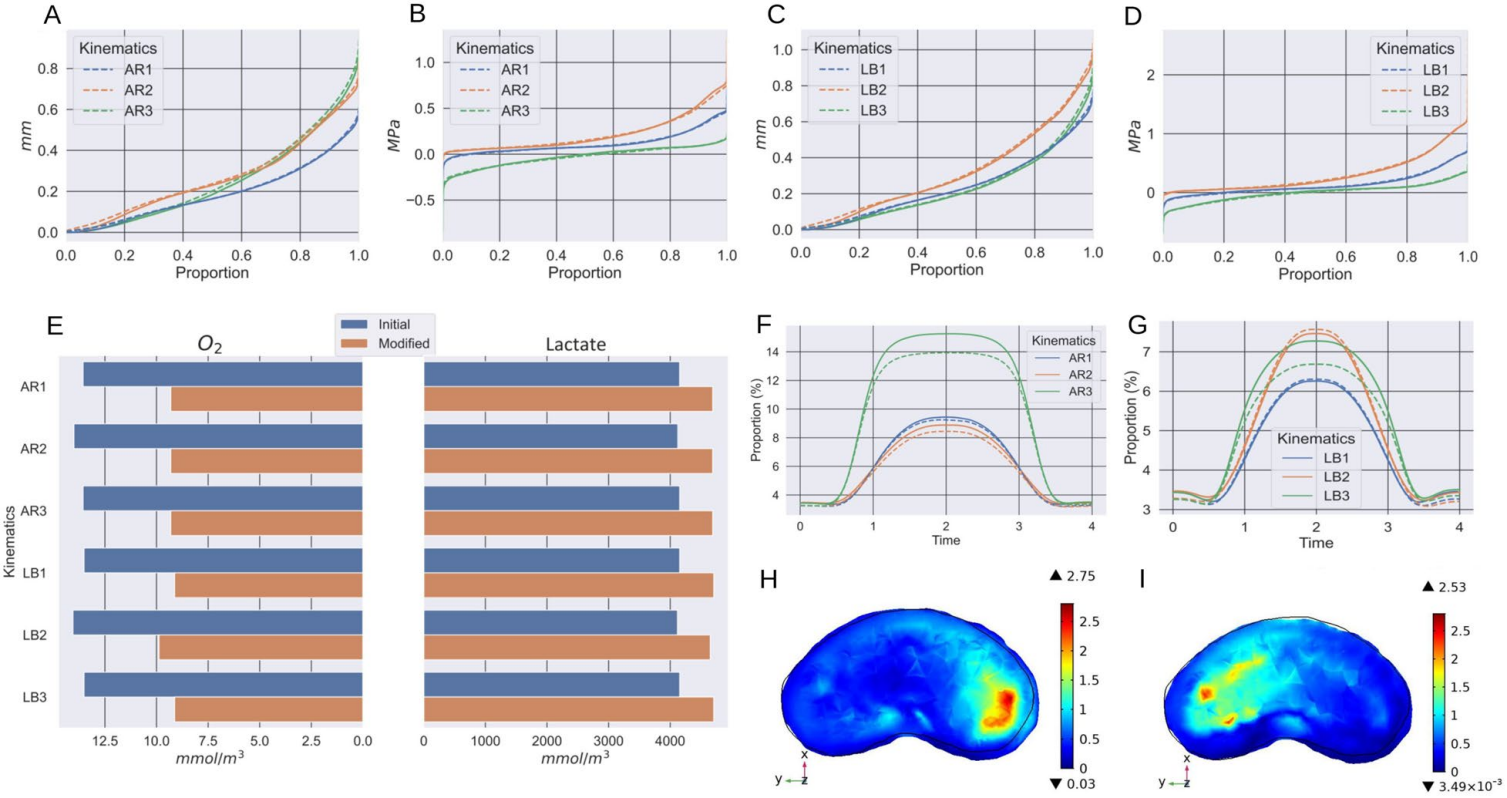
**(A–D)** Empirical cumulative distribution functions of the displacement (**A,C**) and IDP fields (**B,D**). Solid line: initial geometry; dashed line: modified geometry. E: Critical nutrients concentration at $t=2$ s for the initial (blue) and modified (orange) geometry. (**F,G**) Proportion of the anisotropic strain energy with respect to the total strain energy over time during LB (**F**) and AR (**G**). (**H,I**) Ratio of the first eigenvalue of $PK_{aniso}$ over the first eigenvalue of $PK_{iso}$ at $t=2$ s during AR1 (**H**) and AR3 (**I**).

### IDP field

Lateral bending yielded on average higher intradiscal pressure than axial rotation with no notable change between the two considered geometries as depicted by the empirical cumulative distributive functions (Fig. 4B,D). However the kinematics of the ICR drastically impacted the IDP field: the predicted values at the centre of the disc range between $-0.064$ MPa (LB-3) and 0.251 MPa (LB-1) for the initial geometry and between $-0.058$ MPa (LB-3) and 0.249 MPa (LB-1) for the modified geometry during lateral bending. On a more global scale, the median IDP value was the lowest with a mobile ICR (LB3: 0.021 MPa, AR3: 0.02 MPa), followed by an ICR at the centre of the disc (LB1: 0.079 MPa, AR1: 0.078) and finally by an off-centre static ICR (LB2: 0.180 MPa, AR2: 0.142 MPa).

### Displacement field

For a given ICR kinematics, the geometry did not significantly affect the displacement fields as depicted by their empirical cumulative distribution functions (Fig. 4A,C); the relative difference on the maximum of the displacement field between the two geometries was up to 2.8%. More specifically, During LB, the maximum of the displacement field considering a mobile centre of rotation was respectively 0.996 mm and 1.024 mm for respectively the initial and the modified geometry and their location was on the extreme left side of the disc. On the other hand, using a static centre of rotation (LB1, LB2) lead to a maximum of the displacement field in the right side with different magnitudes (LB1: 0.774 mm, LB2: 1.054 mm). Regarding AR, the maxima ranged from 0.600 mm (AR 1) to 0.966 mm (AR3), passing through 0.833 mm (AR2). Its location was on the left side of the disc for the assumptions AR1 and AR3 while it was reported on the right side under hypothesis AR2.

### Nutrients concentration fields

In line with the literature related to the IVD, we define the critical nutrients concentrations as the minimal oxygen ($[O_{2}]^{*}$) and maximal lactate concentrations ($[Lact]^{*}$). For all kinematics scenarios, the modified geometry with the wedge angle had a substantial influence on the critical concentrations when compared to the initial geometry. On average, the minimal oxygen concentration dropped by 32.3% while the maximal lactate concentration increased by 13.4% (Fig. 4E). The ICR kinematics only affected marginally the nutrients fields: considering a static centre of rotation at the disc barycentre (LB1, AR1) or a moving ICR (LB3, AR3) gave the same predictions ($[O_{2}]^{*} =$ 13.51 mmol m$^{-3}$, $[Lact]^{*} =$ 4155 mmol m$^{-3}$ during lateral bending and $[O_{2}]^{*} =$ 13.56 mmol m$^{-3}$, $[Lact]^{*} =$ 4154 mmol m$^{-3}$ during axial rotation). However, when considering the ICR in an off-centre location (LB2, AR2), $[O_{2}]^{*}$ increased on average by 5.2% and $[Lact]^{*}$ increased on average by 1%.

### Anisotropic strain energy

For all the kinematics of interest, the proportion of anisotropic strain energy with respect to the total energy tended to increase with the range of motion (Fig. 4F,G). On average, axial rotation yielded higher proportion (up to 15.3%) than lateral bending. The geometry of the wedge angle had a marginal effect on the maximum of the anisotropic strain energy proportion (an average difference of 3.4% during LB and 5.1% during axial rotation). Spatial analysis of the ratio of the first eigenvalue of $PK_{aniso}$ over the first eigenvalue of $PK_{iso}$ during axial rotation revealed that the fibres in the postero-lateral compartments are the most solicited. However, the right side fibres were more solicited assuming hypothesis AR1 but the left side fibres were the most solicited assuming AR3. In both cases, the maximum of the ratio are comparable (AR1: 2.75, AR3: 2.53).

### Peclet number

The maximum values (i.e. at $t=1$ s) for each definition of the ICR and for the two geometries are gathered in Table 4. The reported values are of the same order of magnitude between the geometries and kinematics. A static centre of rotation at the disc barycentre systematically lead to lower maximal values.

**Table 4:** Peclet number associated with $O_{2}$ and lactate at $t=1$ s for each configuration.

Geometry	Lateral bending						Axial rotation					
	Oxygen			Lactate			Oxygen			Lactate		
	LB-1	LB-2	LB-3	LB-1	LB-2	LB-3	AR-1	AR-2	AR-3	AR-1	AR-2	AR-3
Initial	743	903	817	1604	1950	1628	552	720	886	1193	1554	1913
Modified	694	860	787	1499	1858	1699	531	705	884	1147	1523	1909

## Discussion

The location of the intervertebral instantaneous centre of rotation is a controversial topic. Hence, the purpose of this study was to assess the sensitivity of the main IVD variables to the assumptions made on its centre of rotation kinematics and geometry during lateral bending and axial rotation. The major outcomes are that the kinematics of the centre of rotation considerably affects the pressure and displacement fields as well as the strain energy, while the wedge angle markedly impacts the nutrients’ concentrations.

Porcine models are an opportune alternative to human models and are often used to investigate injury mechanisms, surgical approaches or implant tests; more details and references on this topic can be found elsewhere^34,35^. Lumbar porcine discs are indeed similar to human discs in many ways. First, they have similar dimensions, albeit slightly smaller in the anteroposterior axis (average depth of 24.9 mm for the swines versus 34.6 mm for humans)^36,37^. The anatomy of the lumbar unit, especially regarding the posterior elements, are also akin since no statistically significant differences on the spinous and transverse processes lengths and between-facets dimensions were reported^38^. Then, both have analogous concentrations and gradients in terms of water content (67–83% for humans^39^ versus 71–85% for pigs^40^), proteoglycans content (humans: 5–11 $\upmu$g of uronic acid by mg of wet tissue^39^ versus 1 to 10 in a pig disc^41^) and collagen content (humans: 30–48% of dry weight^42^ versus 20.8–48.6% in pigs discs^40^). Finally, from a chemical aspect, porcine and human discs are closely related as well: the ratio of chondroitin sulfate to keratan sulfate was shown to be similar (1.75–4 for pigs^41^ versus 1.79–2.5 for humans^39^). The main difference that has to be highlighted is the presence of notochordal cells in swine nuclei, even at the adult stage when such cells disappear in human discs during adolescence^43^. If this specificity has to be kept in mind while investigating disc degeneration or therapeutic treatments, it has, to the best of our knowledge, only little to no direct influence on the macroscopic, mechanical response of the disc.

However for the kinematic analysis we aimed to perform in this study, some questions may have arisen as pigs are quadrupeds when humans are bipeds. Previous studies compared human and porcine spines and reported that both spines had similar range of motion during lateral bending and axial rotation^34^: range of motion up to 6$^\circ$ were reported during lateral bending and up to 2$^\circ$ during axial rotation of a swine spine. These values match the upper values of the study of Pearcy and Tibrewal^16^ we used as reference for our simulations (AR: 3$^\circ$, LB: 6$^\circ$). Moreover, in standing situations, the human spine bears the weight of the upper body which is a notable difference with quadrupeds. Despite this distinction, previous work argues that the load exerted on quadrupeds spines are comparable in terms of magnitude and direction to the load put on humans spines, as a result of the muscles forces required for stabilisation^44^. Finally, the parameters (Supplementary Table 1) describing the mechanical behaviour of our porcine model fall in the range of values reported in previous models of human discs^45–47^. We therefore consider our model to be of valuable insights for the study of the human centre of rotation kinematics with the main advantage of having a realistic description, not only of the geometry like many other models, but more importantly of the internal biomechanical variables.

**Figure 5:**
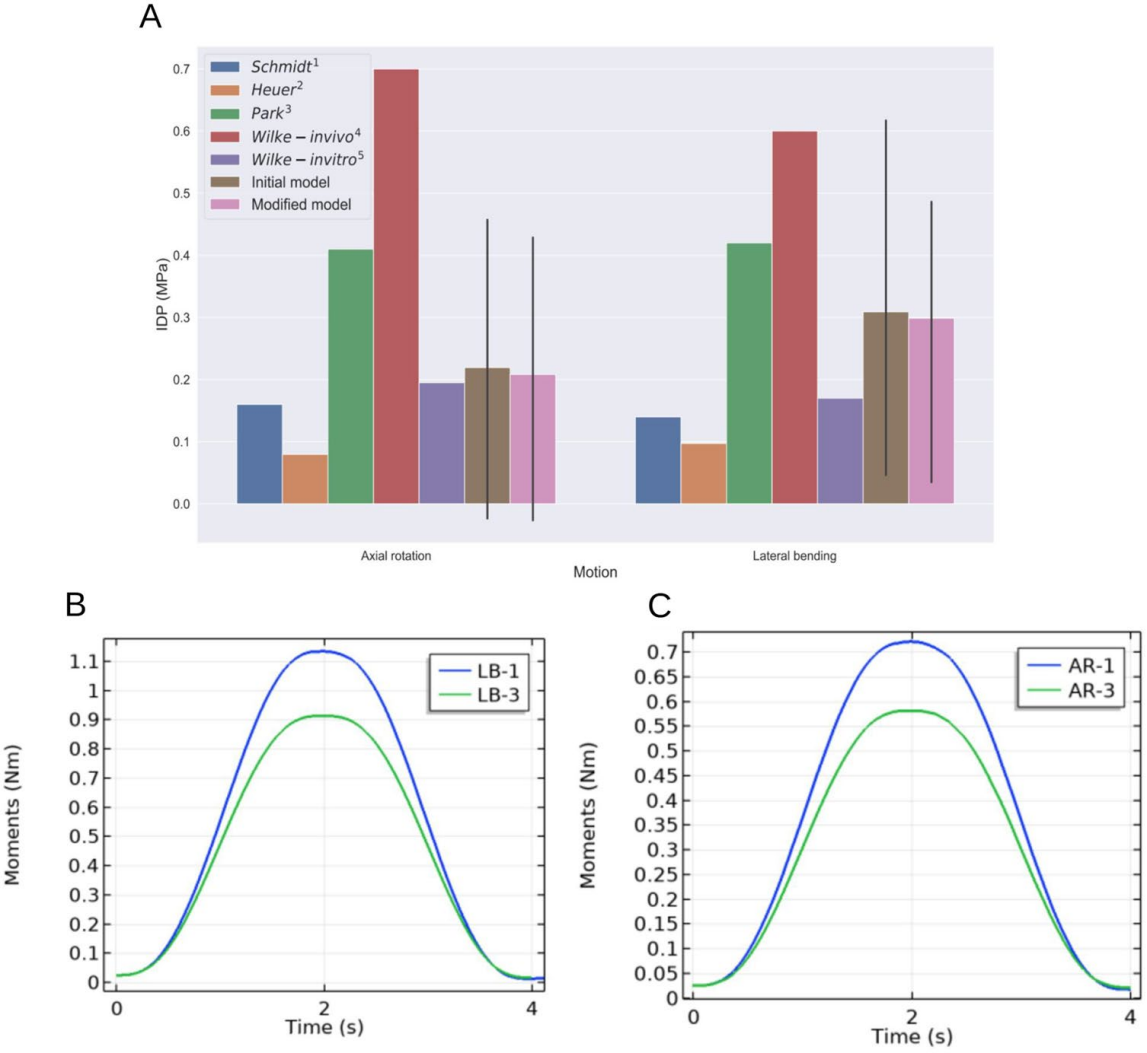
(**A**) Comparison of the predicted nucleus pressure with previous studies. (1) Schmidt et al., 2006^48^, (2) Heuer et al., 2006^49^, (3) Park et al., 2013^50^, (4) Wilke et al., 2001^51^, (5): Wilke et al., 1996^52^. (**B**) Total reaction moment over time during LB considering a static (LB-1) and a mobile (LB-3) ICR and using the initial geometry. (**C**) Total reaction moment over time during AR considering a static (AR-1) and a mobile (AR-3) ICR and using the initial geometry.

Our results can be validated through comparisons with previous works. Range of motion happens to be a preferential validation metric in the literature but as we used it as input through a prescribed displacement, we will instead use the intradiscal pressure. The pressure computed in the initial unloaded state is in good agreement with the in vivo data previously reported by Wilke and colleagues^53^, whose values in the NP range from 0.10 to 0.25 MPa for different kinds of lying positions. As for the specific validation during lateral bending and axial rotation, Fig. 5A displays a comparison of the mean IDP in the nucleus predicted by our simulations with measures or predictions found in the literature. The reported values show large variations that can be explained by the choice of the methodology. First, the age of the specimens used in the reference studies ranges from 26 to 52 which is most likely to span several disc degeneration grades which is known to alter the IDP. Additionally, the experimental conditions were diverse: the magnitude and type of loading differ between papers. Nonetheless, our mean values (0.22 MPa during AR and 0.31 MPa during LB) are close of the values predicted by the model of Schmidt and coworkers^48^ for their combined moment without follower load conditions which can be compared to our simulations. The in vitro tests of Wilke and colleagues^52^ under pure moment solicitation also tend to validate our results. At last, the notable contrast with the in vivo measurements of Wilke^51^ might be explained by the muscles acting on the spine. In his *in vitro* experiment, he indeed showed the significant impact of the muscles on IDP measurements for a L4/L5 disc. Because of inter-subject variability, an accurate prediction of the IDP was proven to be a tough issue: a comparative study showed up to a five-times difference on the prediction between already validated models of the lumbar spines^54^. As our estimations are in the range of the results presented in this above-mentioned comparative study, we consider our model valid.

The predicted maximum lactate concentration of 4.12 mol m$^{-3}$ is below the value (5.45 mol m$^{-3}$) computed by earlier models^55^. However, both the average and maximal lactate concentration compares with the values predicted by a recent study using patient-specific models for a cohort of ten patients without disc-related disorder^33^ and the computed values fall in the range 2–6 mol m$^{-3}$ measured in human discs with different levels of degeneration^56^. As for the oxygen levels, the critical oxygen concentration of 1.36 kPa is slightly above the measured range 0.3–1.1 kPa reported in canine nucleus^57^ but considerably below the average value of 4 kPa predicted by Shalash et al.^33^. As the values measured on human discs during spinal surgery show a significant variability (0.67–20 kPa^56^), our predictions still seem acceptable. Finally, the critical oxygen and lactate concentration are found in the antero-lateral part of the disc, at the boundary between the inner annulus and the nucleus, which is consistent with previous findings^33,55^.

As modeled by the empirical work of Bibby and colleagues^27^, the IVD metabolism is closely linked to the local concentration of oxygen and lactate through its connection to the medium pH. The concentration fields and the critical concentrations are therefore key features whose sensitivity needs to be investigated. Although the critical concentrations stay in the range of values reported in literature, we do report an average drop of 32.3% of the minimal oxygen concentration in the modified geometry with respect to the initial. As our metabolic model is only diffusive and does not account for any advection, this drop is most likely due to the higher diffusion distance from the cartilaginous endplates which are known to be the major nutrients supply pathways^57,58^. In parallel, using the modified geometry, the maximal lactate concentration predicted increases only by 13.4% on average; the relative difference with the drop in oxygen is explained by the different diffusibility of the nutrients. These results highlight the importance of the geometry to compute the nutrients concentration fields. A previous set of studies indeed showed a 3% increase (respectively 22% decrease) in the maximal lactate (respectively minimal oxygen) concentration between a 3D axisymmetric and a 3D realistic model of a lumbar disc^55,59^. The significant impact of the disc size was also corroborated and quantified by a recent study^33^ which performed a sensitivity analysis to a change on the diffusivity, reaction rates or disc size.

Lateral bending and axial rotation are, by definition, non-uniaxial motions. As a consequence, large pressure gradients arose in our simulations which can impact the fluid flows. Even though we chose a motion duration twice as long as in a previous study^15^, we have the same order of magnitude for our Peclet numbers ($10^{0}$ to $10^{3}$) which confirms that the advection should be taken into account when modelling physiological rotations of the spine. However, a spatial analysis using local values of fluid velocity and diffusibility showed significant variations up to 60 times lower than the values reported in Table 4. It appeared that the high global value of the Peclet number was the consequence of the high local fluid velocity at the lateral boundaries of the disc. We therefore acknowledge that our numerical values should be interpreted with care, but as a complete assessment of the impacts of the advective term showed that for LB and AR there are no significant differences on the critical concentrations and only moderate differences on the concentration fields near the borders^60^, we consider our predictions reliable. We also investigated the potential sensitivity of the porosity and osmotic pressure fields as they are the variables driving the water flows. Our dynamic simulations did not show differences between the geometries or the assumptions on the ICR. Although not representative of activities of daily living, we also ran stationary simulations for LB-1, LB-2, AR-1 and AR-2 hypotheses, which showed respectively a 12.5% and 25% increase of the maximal osmotic pressure with respect to the initial state during respectively axial rotation and lateral bending. These differences between stationary and dynamic simulations are likely explained by the diffusion being too slow with respect to the motion duration.

While the predicted IDP and displacement fields from our simulations appear to be fairly insensitive to the wedge angle (Fig. 4A–D), the ICR kinematics strongly altered these fields. For both LB and AR, a mobile ICR yields substantially lower predictions of the IDP. A possible explanation may lie in an underlying pressure optimisation mechanism. This idea was first raised using musculoskeletal models: in a sensitivity study of the lumbar spine kinetics to the position of the ICR during flexion, it was suggested that the ICR is moving to minimize joint reaction forces^12^, which would subsequently minimize the IDP. However the authors only investigated this idea for motion restricted to the sagittal plane and they acknowledged that further work would be needed to conclude on this interpretation. Our results tend to be in agreement with such an hypothesis: with a mobile ICR, the total moment undergone by the disc over time is up to 19% smaller than with a static ICR set at the centre of the disc (Fig. 5B,C). The evolution of the IDP at the centre of the disc also hints in this direction as at the full range of motion the total pressure is on average 0.326 MPa lower with a mobile ICR than with a static ICR at the disc barycentre. On the other hand, a mobile ICR also increases the maximal values of the displacement field up to 28.7% during LB and 61% during AR.

Furthermore, in our simulations a mobile ICR increased the proportion of anisotropic strain energy, especially during axial rotation where it represents up to 15.3% of the total strain energy, with the proportion increasing with the range of motion. In addition, the analysis of the Piola-Khirchhoff stress tensors associated with the isotropic and anisotropic part of the extracellular matrix (ECM) revealed spatial differences in terms of fibres solicitation. First, it showed that in the areas of higher deformation, most of the stress is put on the fibres (Fig. 4H,I). The collagen fibres are therefore considerably solicited in activities of daily life requiring end-range rotations of the spine. Finally, the fibres in the postero-lateral compartments of the IVD sustain the highest stress which is consistent with clinical observation reporting these areas as common places of injuries such as annular tears. On the other hand, the results displayed on Fig. 4F,G point out that for a smaller range of motion, equivalent to the first 0.5 s of our simulations, the anisotropic strain energy proportion stays nearly constant for every conditions. It implies that the isotropic part of the ECM is the first to stretch and once a certain threshold is reached, the anisotropic component of the strain energy quickly rises.

In short, we tested how the different definitions of the ICR kinematics of a L4/L5 lumbar segment reported in the literature impact the main biomechanical variables of the IVD during lateral bending and axial rotation. Previous studies proposed sensitivity analyses but only for sagittal motions and/or using arbitrary, non-physiological shifts of the ICR^11,12^. Additionally, previous works predominantly focused on how the rotations alter the mechanical variables, such as intradiscal pressure, facet forces, fibre strains or joint reaction forces^8,12,19,48,61^ without looking at the nutrients. On the other hand, papers related to nutrients distribution mostly modeled uni-axial compression^32,59,62^. To our knowledge, we are the first to quantify the differences consequent to the assumption made on the ICR kinematics using finite-element simulations accounting for both the nutrients distributions and the mechanical variables.

Our results find clinical applications, notably for the design and implantation of lumbar discs prostheses. Such prostheses are of particular interest as they are a solution to avoid spinal fusion which is known to significantly decrease the range of motion and have noxious effects on adjacent levels. However, a physiological, moving ICR is required to avoid an overload of the other elements of the spine, such as the facets^63^. Hence our simulations can provide some insights to predict the locations with the highest displacement which can be of help for the conception of new devices.

Even though we wanted to be as close as possible to the physiological reality, some limitations have to be acknowledged and will need to be addressed in future work. Since the L4/L5 lumbar unit is the most prone to degeneration, we chose to focus our study on this level. It can be hypothesized that the behaviour of the other lumbar units would qualitatively be close but our results can not be directly transposed without further validation. Additionally, we used a non-degenerate disc which restricts our findings to healthy discs only. Even though porcine model are appropriate for the study of the spine, future work would greatly benefit from an experimental validation on L4/L5 healthy human discs. For the hypotheses of a moving ICR (LB-3 and AR-3) we used the data reported by Schmidt et al.^9^. However, they computed those trajectories using pure applied moments in the anatomical planes while we made the choice to model the coupled rotations described in the in vivo studies. Nonetheless, the ligaments and posterior elements of their model generate reaction moments and consequently create coupled motion, qualitatively similar to our simulations. The trajectories of the ICR are also constrained in an anatomical plane (frontal for LB and transverse for AR) which is undoubtedly an approximation that does not hold in reality. Further work could focus on more accurate, three-dimensional descriptions of the centre of rotation or alternative ways to model the motion, such as finite helical axis^64^. Finally, it could also be argued that our model lacks of anatomical completeness but as we modeled the same motion for all conditions, it should not bias results. Musculoskeletal simulations entailing more kinematically relevant anatomy (e.g. with muscles and ligaments) could also be of interest and bring more insights on the ICR location.

## Conclusion

We showed manifest evidence that the instantaneous centre of rotation (ICR) kinematics considerably tailor the IVD mechanical response during lateral bending and axial rotation, which emphasizes the importance of patient-specific modeling. Most notably, our work showed that a mobile ICR decreases the intradiscal pressure while increasing the maximal displacement and the stress put on the collagen fibres. On the contrary, it has marginal effects on the nutrients critical concentration; hence an average centre of rotation is accurate enough for nutrients-related simulations but not for the prediction of the displacement and pressure fields. Nonetheless, our results only relate to the L4/L5 level and could benefit from further experimental validation on human healthy discs. Consequently, this work brings new insights on the IVD biomechanics which can notably be of help for the creation of new models or the modeling and design of surgical materials.

## Supplementary Information

Supplementary Information.

## Data Availability

Upon reasonable request to roman.allais@centrale-marseille.fr.
