# Supplementary material for: Biomechanical consequences of the intervertebral disc centre of rotation kinematics during lateral bending and axial rotation
Source: Sci Rep. 2023 Feb 23;13:3172. doi: 10.1038/s41598-023-29551-7 (PMC9950088; doi:10.1038/s41598-023-29551-7)
Supplement: Supplementary file 1 — Supplementary Information. [file 41598_2023_29551_MOESM1_ESM.pdf]

Supplementary information 1: Summary of the model properties

The strain energy density was defined as the sum of a volumetric, an isochoric isotropic and two isochoric anisotropic components (*i.e.* one per fibers family):

$$W = W_{vol} + W_{iso-iso} + W_{aniso,1} + W_{aniso,2}$$

With κ the bulk modulus, μ the shear modulus, I_1 the first invariant of the incompressible part of the right Cauchy–Green deformation tensor, a_i, b_i stiffness and non-linearity coefficients of the family fiber i and I_{2i+2} the orientation invariant associated with the family fibers i.

$$W_{vol} = \frac{\kappa}{2}(J-1)^2$$

$$W_{iso-iso} = \frac{\mu}{2}(I_1 - 3)$$

$$W_{aniso,i} = \frac{a_i}{2b_i}(e^{b_i(\rho(I_{2i+2}-1)^2+(1-\rho)(I_1-3)^2)} - 1)$$

The expression of the osmotic pressure is as follows, with T the absolute temperature, R the universal gas constant, ψ_i and ψ_e osmotic constants, C_e the salt concentration in the external solution and C_{fc} the fixed charge density. The latter is computed using the initial fixed charge density, the initial porosity ϕ_0 and the porosity ϕ.

$$\Pi = RT(\psi_i\sqrt{C_{fc}^2 + 4C_e^2} - 2\psi_e C_e)$$

$$C_{fc} = C_{fc0}\frac{\phi}{\phi_0 J}$$

The permeability k is computed using an empirical relationship[65], using the initial porosity ϕ_0 and current porosity ϕ:

$$k = k_0\left(\frac{e(1+e_0)}{e_0(1+e)}\right)^2 e^{M(\frac{1+e}{1+e_0})}$$

With:

$$e = \frac{\phi}{1-\phi} \qquad and \qquad e_0 = \frac{\phi_0}{1-\phi_0}$$

Supplementary Table 1. Main properties in each subpart of the IVD

Subpart	μ (MPa)	κ (MPa)	a_f (MPa)	b_f (-)	α (°)	k_0 $(m^4/(N.s)$	M (-)	C_{fc0} (mol/m^3)
NP	0.46	0.16	0.41	47.71	-	$0.88e^{-15}$	4.13	250
AF	1.27	1.03	1.49	39.82	30.71	$0.64e^{-15}$	5.61	150
CEP	12.4	5.45	0	1	-	$7.82e^{-15}$	5.34	90

A comparison of these parameters with other models from humans can be found in Chetoui *et al,* 2018[22].
